# Management of femoral neck fractures in young adults

**DOI:** 10.4103/0019-5413.38574

**Published:** 2008

**Authors:** Thuan V Ly, Marc F Swiontkowski

**Affiliations:** Department of Orthopedic Surgery, University of Minnesota, Minneapolis, MN, USA

**Keywords:** Femoral neck fracture, young adult

## Abstract

Femoral neck fractures in young adults are uncommon and often the result of high-energy trauma. They are associated with higher incidences of femoral head osteonecrosis and nonunion. Multiple factors can play a significant role in preventing these devastating complications and contribute to a good outcome. While achieving an anatomic reduction and stable internal fixation are imperative, other treatment variables, such as time to surgery, the role of capsulotomy and the fixation methods remain debatable. Open reduction and internal fixation through a Watson-Jones exposure is the recommended approach. Definitive fixation can be accomplished with three cannulated or noncannulated cancellous screws. Capsulotomy in femoral neck fractures remains a controversial issue and the practice varies by trauma program, region and country. Until there is conclusive data (i.e. prospective and controlled) we recommend performing a capsulotomy. The data available is inconclusive on whether this fracture should be operated emergently, urgently or can wait until the next day. Until there is conclusive data available, we recommend that surgery should be done on an urgent basis. The key factors in treating femoral neck fractures should include early diagnosis, early surgery, anatomic reduction, capsular decompression and stable internal fixation.

## INTRODUCTION

Intracapsular femoral neck fractures are commonly seen in the elderly population after a trivial fall.^1^ However, femoral neck fractures in adults younger than age 50 years are uncommon and often the result of high-energy trauma.^2^–^4^ They account for only 2-3% of all femoral neck fractures.^2^,^5^ To evaluate and treat femoral neck fractures in young adults, it is important to understand and contrast the differences between elderly and young adult patients. Characteristic differences are seen with respect to the osseous and vascular anatomy, the mechanism of injury, associated injuries, fracture pattern and the goals of treatment.

Femoral neck fractures in young adults are associated with higher incidences of femoral head osteonecrosis^4^,^6^–^13^ and nonunion.^4^,^6^,^9^,^14^ The rate of osteonecrosis reported in the literature ranges from 12-86% in young patients after femoral neck fracture.^3^,^4^,^8^,^9^–^16^ This devastating complication may lead to collapse of the femoral head and subsequent osteoarthritis. Reoperation and salvage procedures such as osteotomy have high failure rates and arthroplasty procedures are not ideal given the young age and higher levels of activity.^17^ While achieving an anatomic reduction and stable internal fixation are imperative, other treatment variables, such as time to surgery, the role of capsulotomy and the fixation methods remain debatable. Knowledge of these treatment options and potential complications are beneficial in understanding and managing femoral neck fractures in young adults.

### Anatomy

The blood supply of the femoral head comes from three main sources; the medial femoral circumflex artery (MFCA), lateral femoral circumflex artery (LFCA) and the obturator artery.^18^–^21^ In the adult, the obturator artery provides little and variable amount of blood supply to the femoral head via the ligamentous teres. The LFCA gives rise to the inferior metaphyseal artery by way of the ascending branch and provides the majority of the infero-anterior femoral head. The largest contributor to the femoral head, especially the superolateral aspect of the femoral head is the MFCA.^21^ The lateral epiphyseal artery complex comes from the MFCA and courses along the posterosuperior aspect of the femoral neck before supplying the femoral head. It is important to know and understand that these terminal branches supplying the femoral head are intracapsular. Thus, disruption or distortion due to fracture displacement of terminal branches to the femoral head plays a significant role in the development of osteonecrosis.^22^–^25^ Variables that have been hypothesized in contributing to femoral head osteonecrosis include vascular damage from the initial femoral neck fracture,^8^,^4^,^13^,^26^,^27^ the quality of reduction or fixation of the fracture (restoring flow to the distorted arteries)^8^,^4^,^11^,^13^,^28^ and the elevated intracapsular pressure.^25^,^29^–^34^

### Diagnosis

In the elderly patients, femoral neck fractures usually occur as a result of a fall from standing height. Poor bone density, multiple medical problems and propensity to fall are major risk factors for femoral neck fracture. In young adults, the mechanism of injury is often high-energy trauma, such as motor vehicle accident or fall from height. Fractures that occur in this normal bone density population require substantial axial load with the hip in an abducted position.^4^,^8^ The clinical evaluation of these patients requires a thorough trauma workup because they frequently have other associated injuries.^6^,^8^,^15^,^35^ Despite this, diagnosis and treatment of femoral neck fractures in young adults should only be superseded by other life and limb-threatening injuries. The clinical presentation of patient with femoral neck fracture will show a shortened, flexed and externally rotated leg. Radiographic evaluation should include antero-posterior (AP) pelvis, AP and lateral plain radiographs of the entire femur. Although uncommon, ipsilateral femoral neck and shaft fractures can occur in 2-6% of all femoral shaft fractures. 36–42 These concomitant injuries can be challenging to reduce and the best methods of fixation are debatable.

The fracture pattern seen in young adults will be different from the elderly patients. The poor bone quality and fall from a standing height leads to a low-energy injury and results in a femoral neck or intertrochanteric hip fracture; the femoral neck fracture seen in elderly patients will often be subcapital. It is common to see a transverse fracture pattern with impaction at the fracture site. The fracture pattern seen in young adults will be significantly different because of their better bone quality and higher energy mechanism. The axially loaded mechanism onto an abducted hip will often result in a basicervical or more distal neck fracture; the fracture pattern has a tendency to be more vertically oriented and thus is biomechanically more unstable.^43^–^47^ These characteristics have important implications in terms of obtaining and maintaining stable fixation to allow healing to occur.

**Figure 1:**
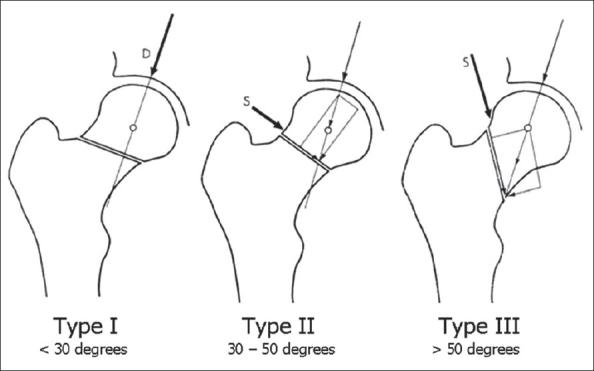
Pauwels' classification (Modified with permission from Bartonicek J. Pauwels' classification of femoral neck fractures: Correct interpretation of the original. *J Orthop Trauma.* 2001;15: 358-360)

Despite known limitations, femoral neck fractures in elderly patients are frequently described using the Garden classification.^48^,^49^ In this age group, treatment can be recommended based on describing the fracture as nondisplaced (Grade I, II) or displaced (Grade III, IV). The Garden classification is not as useful for describing femoral neck fractures in young adults. Pauwels' classification^43^ [Figure 1] might be more descriptive and useful because it is based on fracture pattern and is concerned for achieving a stable fixation in femoral neck fracture in the young population. Pauwels' classification is based on the angle of femoral neck fracture relative to the horizontal axis. They are as follows: Type I: <30 degrees relatively horizontal, Type II: 30-50 degrees, Type III: >50 degrees. This biomechanical model implied that Type I femoral neck fracture will have more instrinsic stability because of the compressive forces that predominate. On the other end of the spectrum, Type III femoral neck fractures are more unstable and seen in young adults more frequently. The fracture pattern is more vertically oriented, resulting in increased shear force, varus moment and instability. Type III fracture patterns have been shown to be more difficult to achieve fixation and have higher risk of fixation failure, malunion, nonunion and osteonecrosis.^43^–^47^

### Management concepts

The goals of treatment are different for the elderly versus the young adults. In the elderly patient, the goals are mobility with weight bearing as tolerated and minimizing complications seen with prolonged bed-rest. Multiple surgical options are considered; reduction and internal fixation, hemiarthroplasty or total hip arthroplasty. Considerations include the patient's physiological age, level of activity, medical comorbidities and the degree of bone density.

In the young adult there is really only one treatment option and that is to do an open reduction and internal fixation of the femoral neck fracture. The main goals are to preserve the femoral head, avoid osteonecrosis and avoid nonunion. Arthroplasty procedures are not ideal given the younger age and high functional levels. Anatomic reduction and stable internal fixation is paramount for a good outcome, but other treatment issues such as closed versus open reduction, the role of capsulotomy and the time to surgery remain controversial. Fixation methods differ, but this is a less controversial topic.

### Surgical approach

There is good agreement that after life and limb-threatening injuries have been addressed if the patient is hemodynamically stable, surgical fixation of the femoral neck should proceed expeditiously. The injured limb should be left shortened and externally rotated while waiting for surgery. Temporary reduction of the femoral neck fracture by extension and internally rotating the limb should be avoided. Several authors^27^,^30^,^34^,^50^ have shown that the intracapsular pressure changes with hip position in femoral neck fracture. Intracapsular pressure is highest when the hip is in extension with internal rotation and decreases significantly when the hip is in flexion with external rotation.

The surgery can be performed with the patient positioned on a fracture table with traction or on a radiolucent flat top table with the leg draped free and no traction. Closed reduction can be attempted by flexing the hip to 45 degrees with slight abduction. This is followed by extending and internally rotating the leg while applying longitudinal traction. Anatomic reduction should be visualized on the fluoroscopic imaging before considering percutaneous fixation. There should be a low threshold to proceed with an open reduction and internal fixation if there is any question about the reduction.^51^,^52^ We recommend that surgery should be done with the patient in supine position, on a radiolucent table and the leg draped free. This positioning will allow you or other surgical teams to address associated injuries, ease of imaging and good visualization for reducing the femoral neck fracture.

**Figure 2:**
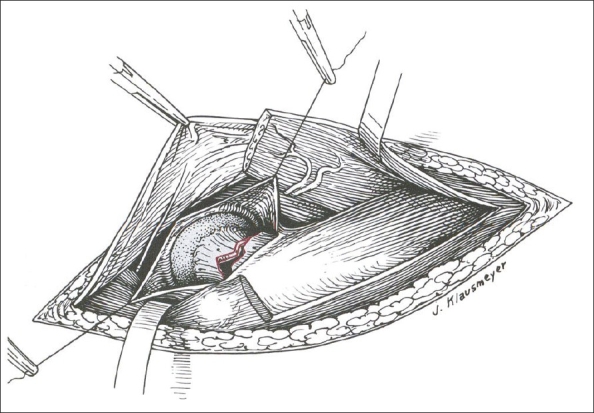
The Watson-Jones anterolateral exposure to the hip for open reduction of femoral neck fractures. The interval between the tensor fascia and gluteus medius is exposed. T-capsulotomy and visualization of the femoral neck fracture (Permission from Swiontkowski MF. Intracapsular Hip Fractures. In Browner BD, Jupiter JB, Levine AM, Trafton PG, editor: Skeletal Trauma, Basic Science, Management and Reconstruction, 3^rd^ ed. Philadelphia; Saunders; 2003, pp.1735)

Surgery is accomplished through the Watson-Jones approach^53^,^54^ [Figure 2]. A straight lateral incision is made over the lateral proximal femur. The incision is curved anteriorly in the proximal portion toward the gluteal pillar of the ilium. The interval is between the tensor fascia and gluteus medius. The tensor fascia is retracted anteriorly and the gluteus medius is retracted posteriorly. The pericapsular fat needs to be swept off to visualize the anterior hip capsule. One can elevate a little bit of the vastus lateralis off the greater trochanteric ridge for further visualization. A T-capsulotomy, with release of the capsule of the intertrochanteric ridge, is performed in line with the femoral neck. This allows for decompression of the hematoma and direct visualization of the femoral neck fracture. The edges of the capsule can be tagged with no. 1 nonabsorbable suture for retraction. Inserting a small, pointed Hohmann retractor extracapsularly onto the anterior part of the acetabular rim can help in better visualization.

For the reduction, a bone-hook or a 5 mm Schanz pin can be used on the distal segment of the fracture. The bone-hook can be placed onto the greater trochanter for lateral traction and the leg can be manipulated into external rotation of leg. This will disimpact the fracture and facilitate reduction with an internal rotation maneuver. The alternative is placing an anterior to posterior Schanz pin several centimeters distal to the fracture site and using this to manipulate the fragments. For the proximal segment, 2-0 mm Kirschner wires can be placed into the femoral head and used as joysticks to lift the proximal fragment anteriorly and reduce the fracture. Once the femoral neck fracture is anatomically reduced by direct visualization of the anterior cortex and by flourscopic imaging, then a Weber clamp or 2-0 mm Kirschner wires can provisionally hold the reduction.

**Figure 3:**
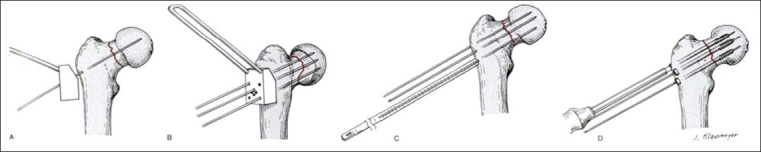
Internal fixation of a femoral neck fracture with a cannulated screw system. A, B, Reduction is confirmed, three parallel guide wires are placed using the guide and fluoroscopic control. C, Length of the wires is measured. D, Screws are inserted over the guide wires to the preselected depth (Permission from Swiontkowski MF. Intracapsular Hip Fractures. In Browner BD, Jupiter JB, Levine AM, Trafton PG, editor: Skeletal Trauma, Basic Science, Management and Reconstruction, 3^rd^ ed. Philadelphia; Saunders; 2003, pp.1737)

Definitive fixation can be accomplished with three cannulated or noncannulated cancellous screws [Figure 3]. Closure is performed with reapproximating the capsule loosely, followed by layered closure with interrupted absorbable sutures and nylon or staples for the skin. A hemovac drain should be placed before the fascial closure.

Postoperative regimen should include postoperative antibiotics for 24 h, deep venous thrombosis prophylaxis with low molecular weight heparin or coumadin for four to six weeks (depending on patient mobility) and physical therapy consultation. The toe-touch weight-bearing with crutches or a walker were allowed for at least eight weeks. Increased weight bearing can be allowed based on the healing seen on routine monthly radiographic follow-ups. If there is evidence of healing at eight weeks, patients are allowed to begin partial weight bearing (up to 50% of body weight) with crutches or walker. Full weight bearing is allowed at 12 weeks. Patients are instructed to wean off the crutches when they are able to ambulate without a significant limp.

### Fixation methods

Multiple clinical and biomechanical studies have evaluated the type and number of implants (cancellous screws) for treatment of femoral neck fractures.^44^–^47^,^55^,^56^ The limitations of these studies are that all of these studies are based on osteoporotic bone models. However, the basic biomechanical principle should still be able to be applied to young adults with good bone density. For most femoral neck fractures, the recommended fixation technique is with multiple cancellous lag screws [Figures 4A and 4B]. The Pauwels' Type I and II are most amenable to this type of fixation. These fracture patterns allow for parallel placement of three cannulated screws in an inverted triangle configuration and perpendicular placement of the screws to the fracture line to allow for optimal compression across the fracture site. The use of a fourth screw has not been shown to have a significant increase in mechanical advantage in most femoral neck fractures.^57^ However, in femoral neck fractures with posterior comminution, a fourth screw would be beneficial.^56^

**Figure 4A:**
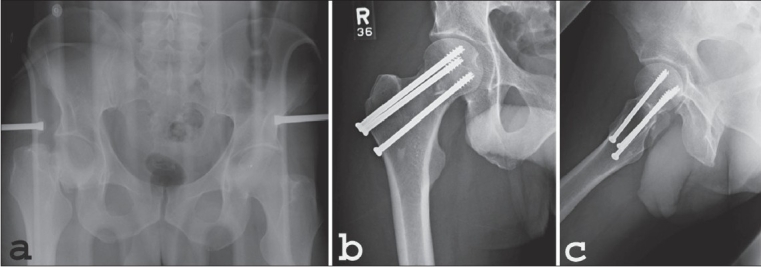
A: AP radiograph of pelvis (a) in a 22 y/o male shows displaced right femoral neck fracture following a motor vehicle accident. (b) Postoperative AP radiograph (c) Lateral hip radiographs after open reduction and internal fixation with three cannulated cancellous screws

**Figure 4B:**
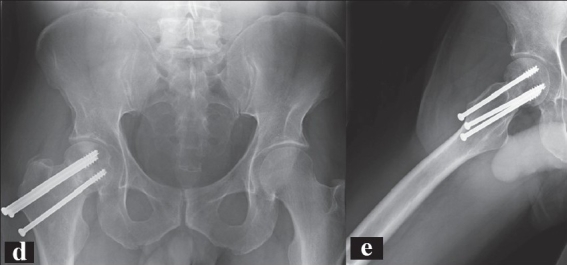
B: AP radiograph (d) and lateral radiograph (e) of the same patient at 8 month followup. There is some settling of the fracture along with the comminution and a lack of complete healing at this point. Functionally, the patient has no pain and is full weight bearing.

The sliding hip screw (SHS) is an alternative to consider.^45^,^46^,^55^ Basicervical femoral neck fractures with comminution is a fracture pattern where SHS will provide more stable fixation than three cancellous screws. Blair *et al*.,^55^ performed a biomechanical cadaver study to evaluate three different fixation techniques for treatment of basicervical femoral neck fracture. They recommended the use of SHS over the use of multiple cancellous screws. Moreover, they found that a derotation screw located superior to the SHS did not add any increase in fixation after the SHS is placed. However, we recommend still using a derotational screw or a second Kirschner wire to prevent rotation of the femoral head during insertion of the compression screw because of the good bone density in a young adults.

Another fracture pattern that would be amenable to SHS is the more vertically oriented femoral neck fracture (Pauwels' Type III). The dominant shear force that is seen with this high-angle fracture pattern lends itself to a higher rate of failure and nonunion.^43^–^47^,^58^ The deformities often seen are varus angulation and inferior translation of the proximal femoral neck/head fragment. Several biomechanical studies^45^,^46^,^55^ have evaluated different implants for managing Pauwels' Type III femoral neck fractures. Baitner *et al*.,^45^ compared multiple screws to the SHS for treatment of Pauwels' Type III femoral neck fracture. They found that the SHS had less inferior femoral head displacement, less shearing displacement and a greater load to failure when compared to the three cannulated cancellous screws. Bonnaire and Weber^46^ looked at four different methods of fixation (SHS with derotational screw, SHS without derotational screw, cancellous screws and a 130 degrees angled blade plate) for Pauwels' Type III cadaveric femoral neck fractures. They concluded that the SHS with the derotational screw is the best implant for this fracture pattern. Routinely using these large compression hip screws does raise concerns about the large amount of bone removed if later reconstruction is required for nonunion, the possibility of damaging the femoral head blood supply if imperfectly placed and their inability to control rotation well without an additional derotational screw.^52^,^59^

The role of concomitant valgus osteotomy and internal fixation of fresh femoral neck fractures have been reported in the literature.^60^–^64^ A valgus osteotomy converts the shear force to compressive forces at the fracture site. This increases the stability of the implant and allows for faster healing. For established femoral neck nonunion, there is good literature to support the method of performing a valgus producing osteotomy as a salvage procedure.^65^–^69^ More recently Magu *et al*.,^60^ reported their outcome on 50 adult patients with osteoporosis who underwent a primary valgus intertrochanteric osteotomy for displaced femoral neck fracture. The interval between injury and surgery ranged from three to 30 days (no mean reported). They concluded that this is a dependable procedure for fresh fractures of femoral neck with osteoporosis.

Our preference for treating Pauwels' Type III fracture is open reduction and internal fixation with three cannulated screws. Obtaining anatomic reduction and adequate fixation remains the key to successful treatment of femoral neck fractures in young adults, as with any other fractures. Failure is often a result of not achieving these principles. This is best accomplished through an open approach to visualize the fracture, anatomically reduce the fracture and compress it with three parallel and optimal placements of the screws. The first screw should be inferior along the calcar, the second should be posterior along the neck and the third should be superior at the tensile surface of the fracture. Postoperatively we delay their time to full weight bearing. They should remain on strict toe-touch weight bearing for a total of 12 weeks and advance weight bearing thereafter.

### Muscle pedicle bone grafting

Muscle pedicle bone grafting has been reported in the literature as an addition to open reduction and internal fixation of femoral neck fractures.^70^–^72^ Meyers *et al*.,^71^ noted that there was a large defect in the posterior neck of the femur in greater than 70% of the cases from their series. The use of a quadratus femoris muscle pedicle graft provides blood supply to the femoral head, structural bone graft to buttress the posterior femoral neck comminution and enhance stability. The reported rate of nonunion was 10% and of femoral head osteonecrosis was 5%. Subsequently, Johnson and Brock^72^ and Morwessel and Evarts^70^ were unable to duplicate the results reported by Meyers *et al*.^71^ We do not use muscle pedicle bone grafting in acute femoral neck fractures. This adjunctive procedure requires the patient to be placed in a prone position. Often, this is not possible because of multiple injuries and requirement of immediate spine clearance. Other concerns include the extensive dissection required and risk of injuring the medial femoral circumflex artery.

### The role of capsulotomy

Capsulotomy in femoral neck fractures remains a controversial issue and the practice varies by trauma program, region and country. There are both animal and clinical studies showing the benefit of capsulotomy. Animal studies^25^,^29^ have shown that increased hip intracapsular pressure results in a tamponade effect and may reduce blood flow to the femoral head. Clinical studies^30^–^34^ show that decompressing the intracapsular hematoma via capsulotomy or aspiration reduces the intracapsular pressures. This decrease in the intracapsular pressure results in improved blood flow to the femoral head and may reduce femoral head ischemia.^25^,^29^,^31^,^32^,^34^ Most of these studies are small series, single-center and uncontrolled.

Bonnaire *et al*.,^30^ reported that 75% of the patients in their study had an increased intraarticular pressure because of hemarthrosis. They believed that an increase in joint pressure was associated with reduced perfusion of the femoral head. Harper *et al*.,^31^ advocated that aspiration of intracapsular hematoma led to increased femoral head blood flow. They used a transducer to measure intraosseous pressure and to quantify blood flow. They performed measurements of the intracapsular pressure in 33 intracapsular hip fractures. The results showed that aspiration of the hematoma led to a significant decrease in intraosseous pressure and an increase in pulse perfusion pressure within the femoral head. They suggested that there is an increase in femoral head blood flow initiated by relieving the tamponade. Stromqvist *et al*.^34^ and Holmberg and Dalen^32^ evaluated intracapsular pressure and its effect on femoral head circulation with Tc-MDP scintimetry. Stromqvist *et al*.,^34^ showed that there is an increase in the uptake of the femoral head after aspiration of the hematoma in femoral neck fracture. Holmberg and Dalen^32^ reported that four out of nine patients had intracapsular pressure greater than 80 mm Hg associated with a low scintimetric rate, which indicated a decreased blood flow to the femoral head. These studies suggested that intracapsular distention of the hip may be one reason for femoral head osteonecrosis. They recommended further studies to determine if performing a capsulotomy would decrease the rate of osteonecrosis.

Other studies,^27^,^73^ however, do not support the concept of increased intracapsular pressure as a major factor for the development of osteonecrosis. Maruenda *et al*.,^27^ performed preoperative intracapsular pressure measurement in 34 patients and followed them for an average of seven years after internal fixation of their femoral neck fractures. They found that five of the six patients who developed femoral head osteonecrosis actually had an intracapsular pressure below their diastolic blood pressure. They suggested that it may be the vascular damage that occurred at the time of injury and not the tamponade effect that resulted in osteonecrosis.

Other variables hypothesized to be related to osteonecrosis include the amount of initial fracture displacement,^4^,^8^,^13^ disruption of the blood supply at the time of fracture,^26^,^27^ the quality of fracture reduction or post-reduction malalignment,^8^,^4^,^11^,^13^,^28^ time between fracture and reduction,^4^,^8^,^11^,^74^,^75^ postoperative time to full weight bearing status,^28^,^76^ fracture nonunion,^4^,^12^,^13^ loss of fracture reduction^11^ and associated ipsilateral femoral neck and shaft fractures. 36–41,77 No solid evidence has been presented as to which factor or combination of factors place the patient at a greater risk of femoral head osteonecrosis.

The randomized controlled trials with sufficient sample size to draw a definitive conclusion on whether or not capsulotomy should be performed are lacking. Table 1 shows a summary of the available literature on femoral neck fractures in young adults with the rate of femoral head osteonecrosis and the relationship with capsulotomy.

**Table 1:** Summary of literature on femoral neck fractures in young adults. The number of osteonecrosis cases are reported and whether capsulotomy was performed

Authors	Year	No. of patients	Osteonecrosis	Capsulotomy
Protzman	1976	22	19	Not reported
Kofoed	1982	17	7	0
Swiontkowski	1984	27	5	17
Tooke	1985	32	6	Not reported
Visuri	1988	12	5	2
Sbih	1989	121	32	Not reported
Gerber	1993	54	5	47
Robinson	1995	46	8	0
Gautam	1998	25	3	25
Jam	2002	38	6	1 (aspiration)
Lee	2003	42	10	3
Upadhyay	2004	48 (CRlF)	7	0
		44 (ORlF)	8	44
Haidukewych	2004	73	17	22
Total		601	138 (23%)	

The conclusion here on the role of capsulotomy is that until there is conclusive data (i.e. prospective and controlled) we recommend performing a capsulotomy. It is easy to perform, adds minimal time and risks to the procedure. Most important it may help that small subset of patients who will develop osteonecrosis of the femoral head. The pooled evidence would indicate that intracapsular pressure plays a role in approximately 15% of patients.

### Timing of surgery

The timing of surgery for femoral neck fractures remains a controversial topic. The data available is inconclusive on whether this fracture should be operated emergently, urgently or can wait until the next day. Advocates of early surgery suggest that the main advantages of prompt reduction of displaced femoral neck fractures are unkinking the vessels and performing an intracapsular decompression to remove the offending agent of increased intracapsular pressure.^8^,^18^,^78^ This will improve and restore blood flow to the femoral head, thus minimizing the risk of femoral head osteonecrosis.^20^,^29^,^31^,^32^,^34^ Swiontkowski *et al*.,^8^ had previously recommended that treatment of femoral neck fracture should be performed emergently within 8 h after injury. Other studies have also confirmed that early surgery (within 6-12 h) can decrease the rate of femoral head osteonecrosis.^5^,^11^,^74^,^75^,^79^

Jain *et al*.,^76^ retrospectively reviewed and compared early (< 12 h) and delayed (>12 h) fixation of subcapital hip fracture in 38 patients. The subjects were 60 years of age or less and the average age was 46.4 years. Radiographic evidence of osteonecrosis developed in 16% of the patients and they were all in the delayed fixation group. Only one out of thirty eight patients had aspiration of the intracapsular hematoma. Age, fracture displacement and method of fracture fixation did not influence the development of osteonecrosis. Using the SF-36 and the WOMAC, they did not find a difference in the functional results between the patients who developed osteonecrosis and the patients who did not have osteonecrosis. They concluded that delayed treatment had an increased rate of osteonecrosis, but did not affect the functional outcome.

On the contrary several studies have reported no differences in the rate of osteonecrosis with delayed surgery greater than 24 h. Haidukewych *et al*.,^13^ retrospectively reviewed 83 femoral neck fractures in patients between the ages of 15-50 years. Osteonecrosis occurred in 23%. They reported that 13 of 53 (25%) femoral neck fractures that were treated within 24 h of diagnosis developed osteonecrosis. Four of 20 (20%) fractures that were internally fixed after 24 h of diagnosis developed osteonecrosis; with the small sample size, the difference was not significant. Upadhyay *et al*.,^14^ performed a prospective, randomized study comparing open reduction and internal fixation (ORIF) and closed reduction and internal fixation (CRIF) in young adults with Garden Grades III and IV femoral neck fractures. One hundred and two patients were randomized with 44 in the ORIF (Watson-Jones approach with a T-shape incision in the capsules) and 48 in the CRIF (Closed reduction and percutaneous pinning). There was no significant difference between the two groups in terms of osteonecrosis (14.6% for the CRIF and 18.2% for the ORIF) at two years follow-up. Risk factors such as age, gender, time to surgery (< 48 h or >48 h) and posterior comminution did not affect the development of osteonecrosis. Most patients in this series were treated more than 48 h after injury.

The multiple factors mentioned above makes it difficult to come to a final conclusion. There are multiple articles that have specifically evaluated the influence of time to reduction and fixation on the outcome. Once again until the results of randomized trials are available, we recommend that surgery should be done on a urgent basis. This implies that the ORIF of the femoral neck should be performed as soon as the patient is considered stable and cleared to undergo anesthesia. Urgent operation allows early reduction, capsular decompression, restoration of the anatomy and restoration of femoral head vascularity by unkinking the vessels.

### Complications of femoral neck fractures

The two most challenging complications of femoral neck fractures in the young adult to deal with are femoral head osteonecrosis and nonunion. Osteonecrosis in a young patient is a devastating complication because of the limited options as compared to elderly patients with osteonecrosis of the femoral head. Osteonecrosis in the elderly is less likely to be symptomatic because of their lower functional demands and level of activity. Fortunately, total hip replacement is a good option and has consistent good results for the elderly patient with symptomatic osteonecrosis. However, there is no good alternative treatment in the young patient with symptomatic osteonecrosis. Younger age and higher function demands make prosthetic replacement fraught with high complications and should be a last resort. Reconstructive options to preserve the hip include osteotomy to unload the segmental area of femoral head collapse, femoral head core decompression, free vascularized bone grafting, hemi-resurfacing of the femoral head and hip arthrodesis.^80^,^81^ However, the best method for treating this difficult complication of osteonecrosis is prevention. This entails doing everything possible under the surgeon's control to minimize further vascular injury to the femoral head. This includes prompt reduction, intracapsular decompression, anatomic reduction, stable fixation and close monitoring postoperatively for osteonecrosis.

Nonunion is another complication of femoral neck fractures which is difficult to deal with. The rate of nonunion is between 10 and 30%.^4^,^6^,^9^,^14^ Fortunately, there are good surgical options available for this problem. The treatment that has consistent good results is valgus osteotomy. 65–69 The goal of treatment is to create an environment that allows for healing. This means converting the shear force to compressive forces at the fracture site. This is done by performing a valgus-producing intertrochanteric osteotomy. This results in changing the more vertical femoral fracture line to horizontal and thus allowing for compression.

Marti *et al*.,^65^ published the largest series of 50 patients who were treated with a Pauwel abduction osteotomy for femoral neck nonunion. The average age was 53 years and the average follow-up was for 7.1 years. Forty-three of the 50 femoral necks' nonunion healed and all the osteotomies healed. The average hip score for 37 of these 43 healed fractures was 91. The seven femoral necks that did not heal underwent prosthetic replacement. More recently, Anglen^69^ reported his own series of 13 patients who were treated with a valgus intertrochanteric osteotomy for failed fixation of the femoral neck. They were all under the age of 60 years and the average follow-up time was 25 months. All osteotomies healed. Eleven of 13 had good to excellent functional outcome. The two poor outcomes had segmental osteonecrosis and went on to have joint replacement.

## CONCLUSION

Femoral neck fractures in young adults are uncommon. They usually occur as a result of high-energy trauma and patients often have associated injuries. Osteonecrosis of the femoral head and nonunion are the two most common and challenging complications. Initial fracture displacement and disruption of the femoral head blood flow are contributing factors that are out of the surgeon's control. However, there are multiple other factors under the surgeon's control that can minimize and prevent these complications. The key factors in treating femoral neck fractures should include early diagnosis, early surgery, anatomic reduction, capsular decompression and stable internal fixation.
